# Dipyrone as pre-emptive measure in postoperative analgesia after tonsillectomy in children: a systematic review

**DOI:** 10.1016/j.bjorl.2020.12.005

**Published:** 2021-01-02

**Authors:** Maira Isis S. Stangler, João Pedro Neves Lubianca, Jaqueline Neves Lubianca, José Faibes Lubianca Neto

**Affiliations:** a Universidade Federal de Ciências da Saúde de Porto Alegre (UFCSPA), Programa de Pós-Graduação em Pediatria, Porto Alegre, RS, Brazil; b Santa Casa de Misericórdia de Porto Alegre, Hospital da Criança Santo Antônio, Porto Alegre, RS, Brazil; c Universidade Federal do Rio Grande do Sul (UFRGS), Faculdade de Medicina, Porto Alegre, RS, Brazil; d Universidade Federal do Rio Grande do Sul (UFRGS), Faculdade de Medicina, Hospital de Clínicas de Porto Alegre, Departamento de Ginecologia e Obstetrícia, Porto Alegre, RS, Brazil; e Universidade Federal de Ciências da Saúde de Porto Alegre (UFCSPA), Faculdade de Medicina, Departamento de Clínica Cirúrgica, Programa de Pós-Graduação em Pediatria, Porto Alegre, RS, Brazil; f Santa Casa de Misericórdia de Porto Alegre, Hospital da Criança Santo Antônio, Serviço de Otorrinolaringologia, Porto Alegre, RS, Brazil

**Keywords:** Tonsillectomy, Dipyrone, Agranulocytosis, Analgesics, Child

## Abstract

**Introduction:**

Tonsillectomy is the 2nd most common outpatient surgery performed on children in the United States of America. Its main complication is pain, which varies in intensity from moderate to severe. Dipyrone is one of the most widely used painkillers in the postoperative period in children. Its use, however, is controversial in the literature, to the point that it is banned in many countries due to its potential severe adverse effects. Because of this controversy, reviewing the analgesic use of dipyrone in the postoperative period of tonsillectomy in children is essential.

**Objective:**

The aim of this study was to review the analgesic use of dipyrone in the postoperative period of tonsillectomy in children.

**Methods:**

Systematic review of the literature, involving an evaluation of the quality of articles in the databases MEDLINE/Pubmed, EMBASE and Virtual Health Library, selected with a preestablished search strategy. Only studies with a randomised clinical trial design evaluating the use of dipyrone in the postoperative period of tonsillectomy in children were included.

**Results and conclusion:**

Only 2 randomised clinical trials were found. Both compared dipyrone, paracetamol, and placebo. We were unable to carry out a metanalysis because the studies were too heterogenous (dipyrone was used as pre-emptive analgesic in one and only postoperatively in another). The analgesic effect of dipyrone, measured by validated pain scales in childhood, was shown to be superior to placebo and similar to paracetamol. It appears that dipyrone exhibits a profile suitable for use in children. However, the scarcity of randomised clinical trials evaluating its analgesic effect in this age group leads to the conclusion that more well-designed studies are still needed to establish the role of dipyrone in the postoperative period of tonsillectomy in children.

## Introduction

Acute postoperative pain is the common symptom present in surgical patients following the procedure.^1^ Pre-emptive analgesia describes the attempt to control pain in the pre- incisional period. It aims to prevent central hyperexcitability, which tends to increase in the postoperative period.^2^ A recent review of systematic reviews concluded that evidence for the efficacy of various drugs and strategies for managing postoperative pain in children is still inconclusive.^3^ Dipyrone, however, was not evaluated in this review.

Dipyrone is effective in postoperative pain in children. There are 8 clinical trials that have used it alone or in combination with other medications.^4^, ^5^, ^6^, ^7^, ^8^, ^9^, ^10^, ^11^ It is one of the most widely-used analgesics in the postoperative period in several European,^12^, ^13^ African^14^ and Latin American countries.^15^ However, there is concern about its potential for associated anaphylaxis and agranulocytosis.^16^ Its use is banned in more than 20 countries.

The real incidence of these adverse effects, however, is low. In a multicenter study involving more than 1,177 children treated with dipyrone in the postoperative period, the incidence of serious adverse effects was less than 0.3%, with no case of agranulocytosis.^17^ Two studies, one involving several European countries (Germany, Italy and Spain)^18^ and another in the city of Berlin alone,^19^ estimated an incidence of agranulocytosis of 1.1 per million inhabitants/year and 0.96 cases per million inhabitants/year, respectively. In the city of Barcelona, the estimate was 0.56 cases per million inhabitants/year.^20^ The risk of severe complications and the availability of alternatives continue to contraindicate its use in the opinion of some.^21^ Despite the ban in several countries, dipyrone is the most widely used painkiller in the postoperative period for children in Brazil, perhaps due to its low cost and the lack of an injectable form of paracetamol, at least until April 2020.^22^ In terms of oral analgesic medication, in contrast with the preference for paracetamol in different regions of the world,^23^ in our country it is the most widely-used self-medication, as it is sold as an over-the-counter medicine.^24^

Dipyrone has been used for decades in children in the postoperative period of tonsillectomy. This surgery is the 2nd most common outpatient procedure in the USA.^25^, ^26^ Its main complication is pain that can lead to a reduction in oral intake, dehydration and weight loss.^27^ The latest North-American Clinical Practice Guideline on Tonsillectomy contains 2 strong recommendations about analgesia: 1) To use analgesics in the post-operative period and 2) The contraindication in the use of opioids, especially in children under 2.^27^ This latter recommendation is based on the FDA’s warning of the risk of respiratory depression and death using codeine.^28^ In the context of tonsillectomy, intravenous dipyrone could represent an alternative to opioids, where odynophagia with consequent difficulty in swallowing is almost universal.

The aim of this study was to review the analgesic use of dipyrone in the postoperative period of tonsillectomy in children.

## Methods

This study is a systematic review of the literature, which involved the work of two researchers independently evaluating the quality of each article and a third one, in case of non-agreement. The formulation of the research question was based on the PICO strategy.^29^ The following questions guided the bibliographic search: Does dipyrone in post-operative tonsillectomy analgesia in children reduce pain complaints compared to placebo according to validated visual analogue scales?^30^ The search was performed in PubMed, MEDLINE, The Cochrane Library, ClinicalTrials.gov, LILAC, and EMBASE to identify peer-reviewed research, in addition to the grey literature (Google scholar, thesis repositories of the 20 most important universities of Brazil), using separate and combined terms, with the Boolean operator OR and, using the following subject descriptors in health sciences from BIREME (DeCS): dipyrone OR metamizole AND postoperative pain; dipyrone OR metamizole AND postoperative pain AND children, dipyrone OR metamizole AND tonsillectomy, dipyrone OR metamizole AND tonsillectomy AND children, dipyrone AND metamizole AND pre-emptive analgesia, dipyrone OR metamizole AND pre-emptive analgesia AND children. Additionally, the references of the selected articles were reviewed in the search for other relevant publications. The selection of articles was carried out in the months of June/20 to Oct/20.

The inclusion criteria were as follows: studies that addressed the treatment of pain with dipyrone in a pre-emptive way or after the end of surgery in children. This was then refined for studies with a randomized clinical trial design and that dealt exclusively with tonsillectomies, with dipyrone alone or in combination with another drug in one of the comparison groups. There was no time limit for publications, and we included all articles available in full in Portuguese, English, Spanish and German. As exclusion criteria, the following were adopted: publications that exclusively included chronic pain assessment and studies in adults.

**Figure 1:**
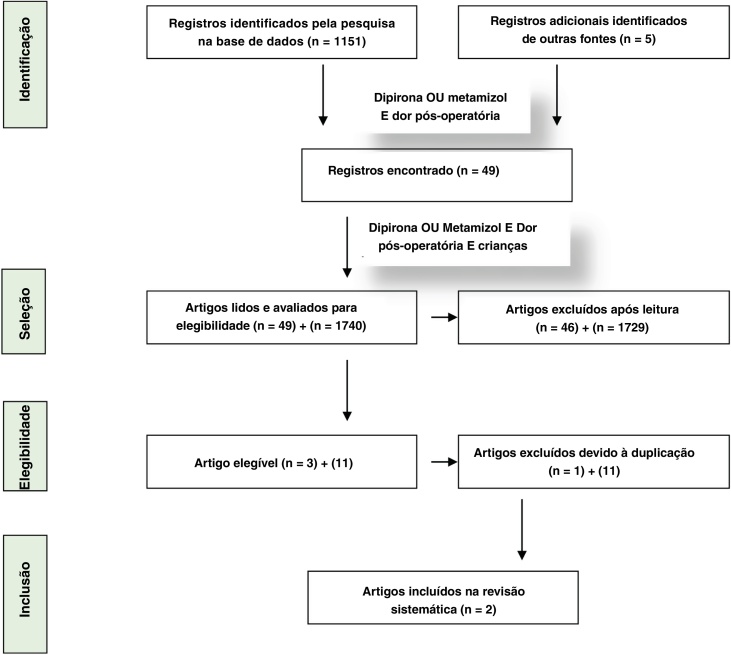
PRISMA flowchart.

The studies were described using the Revised Cochrane Risk-of-Bias tool for randomized trials (RoB2) (Table 1a and b).^31^ The PRISMA strategy (Preferred Reporting Items for Systematic Reviews and Meta-Analysis)^32^ was used in this review in order to qualify the work when performing a critical analysis of the selected studies. Fig. 1 shows the flowchart of identification, selection and inclusion of studies based on the PRISMA recommendation.

**Table 1:** (a and b) Analyses the studies according to ROB 2.

(a) Analysis of the paper of Kocum AI et al. (Intravenous paracetamol and dipyrone for postoperative analgesia after day-case tonsillectomy in children: a prospective, randomized, double blind, placebo controlled study) according to Revised Cochrane risk-of-Bias tool for randomized trials (RoB 2)		
Bias	Author’s judgement	Support for judgement
Random sequence generation	Low risk of Bias	Quote: “…elegible patients were randomized… according to a pre-generated randomization scheme created by the web site Randomization.com. All study medications were prepared by a clinician unaware of the patient’s allocated study group in identical infusion pumps”.
		Comment: a random component was used in the sequence generation process. There are no imbalances that indicate problems with the randomization process. There is no reason to suspect that the enrolling investigator or the participants has knowledge of the forthcoming allocation.
Deviations from intended interventions	Low risk of Bias	Quote: “Patients, all care givers and the clinical observers who scored were blinded to the allocated treatment of the individual patient. Infusions were administered by a blinded attending physician”.
		Comment: the blinding process was well described. There are no cases of non-adherence to the assign intervention regimen also.
Missing outcome data	Low risk of Bias	Quote: “All randomized patients were taken into statistical analysis”.
		Comment: The study population was ideal for analysis of the intention to treat effect since it was composed of 100% of randomized patients.
Measurement of the outcome	Low risk of Bias	Quote: “In case of CHEOPS score > 6 and/or PR score < 2, the patient received 0.25 mg/kg… as rescue analgesic medication until CHEOPS score was ≤6 and PR ≥ 2.” Comment: The measurement instrument (CHEOPS) is a well-known validated scale in children. The rescue pethidine had objective parameters defined a priori to indicate its use, what is also objectively measured. The outcome assessors were blinded to intervention status.
Selective reporting	Low risk of BIAS	Quote: “The number of patients with nausea and vomits, and use of antiemetic medications was similar in the 3 groups” Comment: Improbable, because the outcomes were predetermined and were not multiple analyzed. All the negative and positive outcomes were fully described according to time interval.

(b) Analysis of the paper of Sener M, et al. (Administration of paracetamol vs. dipyrone by intravenous patient-controlled analgesia for postoperative pain relief in children after tonsillectomy) according to Revised Cochrane risk-of-Bias tool for randomized trials (RoB 2).		
Bias	Author’s judgement	Support for judgement
Random sequence generation	Low risk of Bias	Quote: “Patients were randomly assigned to one of three study groups, according to a randomization scheme generated by the website randomization.com (http://www.randomization.com)” “The analgesic solution (500 mL of NaCl at 0, 9%) was prepared by one of the researchers, blinded to the treatment protocol and not involved in the patients’ intraoperative and postoperative treatments”.
		Comment: A random component was used in the sequence generation process. There are no imbalances that indicate problems with the randomization process. There is no reason to suspect that the enrolling investigator or the participants has knowledge of the forthcoming allocation.
Deviations from intended interventions	Low risk of Bias	Quote: “The analgesic solution (500 mL of 0.9% NaCl) was prepared by one of the researchers, blinded to the treatment protocol and not involved in the patients’ intraoperative and postoperative treatments. Patients were also unaware of the treatment. Postoperative data were collected by another anesthesiologist (EC), also blinded to the analgesics used. Comment: the blinding process was well described. There are no cases of non-adherence to the assign intervention regimen also.
Missing outcome data	Low risk of bias	Quote: No specific quote, but the flowchart of the study showed that there were no losses of patients.
		Comment: The study population was ideal for analysis of the intention to treat effect since it was composed of 100% of randomized patients.
Measurement of the outcome	Low risk of Bias	Quote: “Postoperative pain intensity was assessed by the patient according to the horizontal VAS of 0–100 mm in 30 min, 1, 2, 4, 6, 12 and 24 h after the operation. The pain relief score (EAD) was assessed by the patient as: 0 = none, 1 = little, 2 = some, 3 = a lot and 4 = total relief in 30 min, 1, 2, 4, 6, 12 and 24 postoperative hours. Pethidine IV (0.25 mg/kg --- 1) was administered to patients whose EVA score was ≥ 40 mm and/or EAD < 2 and then recorded (the total dose of pethidine was limited to 1.5 mg/kg/6 h).
		Comment: The measurement instrument (CHEOPS) is a well-known validated scale in children. The rescue pethidine had objective parameters defined a priori to indicate its use, what is also objectively measured. The outcome assessors were blinded to intervention status.
Selective reporting	Low risk of Bias	Quote: No specifically quote but reading of method and result sections make it clear.
		Comment: Improbable because the outcomes were predetermined and were not multiple analyzed. All the negative and positive outcomes were fully described according to time interval.

## Results

The maximum number of 1151 articles were selected using the descriptors “dipyrone or metamizol and postoperative pain”. The number of articles found in EMBASE was 1151, in PUBMED 336 and in VHL 345. When the descriptor “children” was introduced to the previous search, the corresponding numbers were 120, 49 and 42. When adding “randomized clinical trial”, the corresponding numbers were 31, 13 and 19. We searched also the grey literature – Google Scholar and Brazilian universities repositories of thesis (1,740 results and 11 duplicates) and found no additional references to be included. The materials and methods of each article obtained from the search with the descriptors “dipyrone or metamizole and postoperative pain and children” were manually reviewed. Nine randomized clinical trials were identified evaluating the postoperative analgesic effect of dipyrone in isolation or associated with one of the groups. When only studies dealing with tonsillectomy were selected, 3 remained and it was seen that there was a duplicate of one. The final search result was 2 randomized clinical trials that evaluated the effect of dipyrone against placebo, one in pre-emptive and the other in the postoperative analgesia, in children undergoing tonsillectomy, with or without adenoidectomy or placement of ventilation tubes.

Table 2, Table 3, Table 4 show information on the 2 articles included. Table 2 shows the study identification, investigational model, follow-up time, inclusion criteria, age, sex, and the sample size.

**Table 2:** Characteristics of selected studies.

Study	Investigational design	Follow-up time	Inclusion criteria	Age	S (M/F)	Sample size
Sener TG, et al. Brazilian Journal of Anesthesiology	Randomised clinical trial	24 h follow-up	7–15 years ASA I tonsillectomy	9.4 ± 2.2	20/20	143 (elect)
				8.8 ± 2.2	20/19	120 (randomised)
				9.2 ± 2.3	20/17	
Kocum AI, et al. Brazilian Journal of Otorhinolaryngology	Randomised clinical trial	6 h follow-up	3–6 years tonsillectomy and/or adenoidectomy and/or grommets ASA I	4.7 ± 10	29/11	138 (elect)
				4.6 ± 10	27/13	120 (randomised)
				4.3 ± 10	19/21	

Elect, elective surgeries patients; randomized, randomized through http://www.randomization.com.

**Table 3:** Descriptions of the studies according to intervention groups, type of analgesia and form of administration of dipyrone.

Study	Intervention groups	Type of analgesia	Form of administration of dipyrone
Sener TG, et al. Brazilian Journal of Anesthesiology	Paracetamol – 40	Postoperative	Paracetamol – 20 mg/mL IV
	Dipyrone – 40		Dipyrone – 20 mg/mL IV
	Placebo – 40		Placebo – 0,9% /1 mL saline IV
	120% – 100%		Paracetamol – 10 mg/kg IV – 4 h
	IV PCA		Dipyrone 10 mg/kg – 4 h 15 mg/kg IV
			PCA
Kocum AI, et al. Brazilian Journal of Otorhinolaryngology	Paracetamol – 40	Pre-emptive (before the induction of general anaesthesia)	Paracetamol – 15 mg/kg IV
	Dipyrone – 40		Dipyrone – 15 mg/kg IV
	Placebo – 40		0,9% / 1 ml NaCl IV
	120%–100%		IV single dose
	IV single doses after anaesthesia induction		

PCA, patient-controlled analgesia.

**Table 4:** Primary and secondary outcomes and main results of the two analysed studies.

Study	Strengths and weaknesses	Primary and secondary outcomes	Main results
Sener TG, et al. Brazilian Journal of Anesthesiology	Inclusion and exclusion criteria: OK	a) Visual Analogue Scale (VAS 0–100 mm ) in 30 min, 1 h, 2 h, 4 h, 6 h, 12 h, 24 h	a) Both active groups significantly better than placebo at 6 h. Dipyrone was also better at 30 min.
	Randomisation: OK		
	Double-blinded: OK	b) Pain relief scores (1–4) 1 h, 2 h, 4 h, 6 h, 12 h, 24 h	b) No difference between groups.
	No sample size calculation prior to allocation (power analysis based on the total pethidine requirement from the first 15 patients)		
		c) Total opioid requirements 30 min, 1 h, 2 h, 4 h, 6 h, 12 h, 24 h	c) Significantly lower with paracetamol and dipyrone groups compared with placebo group (62%, 68.4%, vs. 90%, *p* < 0.05)
		d) Incidence of nausea, vomiting and need for antiemetic medication	d) No difference between groups
	Two-tabled type I error of 0.05 and a power of 80%		

Kocum AI, et al. Brazilian Journal of Otorhinolaryngology	Inclusion and exclusion criteria: OK	a) Pain intensity: Pain scale (CHEOPS – Children’s Hospital East Ontario Pain Scale) with range of scores 4–13. 0.25; 0.5; 1; 2; 4 and 6 after arrival to post anaesthesia care unity	a) No difference in CHEOPS score
	Randomisation: OK		b) No difference between groups at 0.5; 1 h and 2 h - Paracetamol was significantly higher than placebo group at 0.5 and 4 h treatment (*p* = 0.04; *p* = 0.01, respectively) - No differences between dipyrone vs. placebo and dipyrone vs. paracetamol comparisons groups at 5 h of follow-up - At 6 h follow-up pain relief score was significantly higher in paracetamol group when compared to placebo (*p* < 0.001) and dipyrone (*p* = 0.04) groups. Also, at 6 h follow-up pain relief score was significantly higher in dipyrone group than placebo group (*p* = 0.03)
	Double-blinded: OK		
	No sample size calculation prior to allocation (power analysis based on the total pethidine requirement from the first 15 patients)		
		b) Pain Relief: 5-point verbal scale rated by the investigator	
		c) Rescue analgesic medication (pethidine)	
	Statistically significant differences at *p* < 0.05		
			c) Cumulative pethidine use was not different among groups at 0.25; 0.5; 1 h; 2 h and 4 h follow-up. At 6 h follow-up both active groups needed lower rescue analgesic requirement (*p* = 0.01 for paracetamol and *p* = 0.03 for dipyrone)

The studies had short follow-up times (maximum 24 h). While the first study involved only older children, the second study included children from 3 years old. While in the first study, only cases of isolated tonsillectomy were evaluated, in study 2 tonsillectomy either with or without associated adenoidectomy and/or placement of ventilation tubes was allowed.

Table 3 shows the description of the data referring to the intervention groups (random number and number who completed the study in each group), type of analgesia and form of administration of dipyrone. Both studies had a 100% completion rate for the randomized sample. While in the first study analgesia was postoperative, in the 2nd it was applied preemptively, thus, making it difficult to directly compare the two studies. The first study brought an innovation to the pediatric surgery postoperative period, in the use of PCA (patient-controlled analgesia).

It is evident that both studies demonstrated care in their methodological approach. Regarding the pain relief (PR) results measured by validated pain scales in childhood, dipyrone and paracetamol were superior to placebo and similar to each other in many measured intervals. The same was observed for the use of rescue pethidine, and this did not differ between active comparison groups.

## Discussion

### Effectiveness of miscellaneous analgesics with emphasis on dipyrone in the postoperative period of tonsillectomy in children

Dipyrone has an efficacy, measured by the number of patients needed to treat to achieve a 50% reduction in postoperative pain in 4–6 h, that is lower than potassium diclofenac and etoricoxib, but greater than several NSAIDs (paracetamol, naproxen, ibuprofen, celecoxib, aspirin and sodium diclofenac). Unfortunately, all these estimates are for adults, and there are no similar studies in children.^33^ There is one systematic review specifically evaluating the efficacy of dipyrone in postoperative pain in children.^34^ Ten systematic reviews were identified regarding postoperative analgesia in tonsillectomy performed in children. The first studied the use of systemic paracetamol, NSAIDs and opioids. However, it did not comment on dipyrone.^35^ Two analyzed the effect of ketamine via peritonsillar or systemic injection,^36^, ^37^ two studied the effect of corticosteroids,^38^, ^39^ one focused on the effect of bupivacaine^40^ and the other on dexmedetomidine compared to morphine or fentanyl.^41^ A Cochrane systematic review analyzed the form of analgesic prescription (different analgesics, but never dipyrone), if required or fixed.^42^ Finally, there was another review that was restricted to the use of oral rinses and sprays to improve recovery followed by tonsillectomy.^43^ An overview of all these systematic reviews was given in the recent work by Boric et al., 2017.^3^

As demonstrated in the present review, there are only two randomized clinical trials that have evaluated the analgesic effect of dipyrone in the postoperative period of tonsillectomy in children.^6^, ^7^ Unfortunately, it was not possible to perform a meta-analysis, as one study included isolated tonsillectomy^7^ and the other allowed associated adenoidectomy and/or ventilation tubes,^6^ which can lead to different levels of pain, due to the greater manipulation of the patients. In addition, one study evaluated preemptive^6^ and the other postoperative analgesia.^7^ Finally, the study of preemptive analgesia gave a single dose of dipyrone or placebo right after introduction of the anesthetic,^6^ while the other study evaluated analgesia initiated in the postoperative period through the use of PCA.^7^ The limitation is that PCA is not available in most hospitals and requires the understanding and collaboration of the patient, thus being ineffective for young children.

Both studies demonstrated adequate methodological care, with sample size calculations, and with randomization performed through the website randomization.com. Moreover, hypotheses were established a priori, with the use of a placebo with the same characteristics as the active medication, with adequate blinding of both patients and examiners, and with effective and validated outcome measures, in addition to a careful choice of the statistical tests used.

However, the studies have short follow-up times (maximum 24 h). It would be preferable to have a longer follow-up time to monitor the effect of dipyrone, as the pain after tonsillectomy persists for at least 7 days. Another limitation of the results is that, in addition to being developed by the same group of researchers and not being able to be combined for a meta-analysis for the reasons already specified, the data have never been replicated elsewhere by different researchers, which can limit the extrapolation of data to populations other than Turkish patients.

### Agranulocytosis

Agranulocytosis is defined as an absolute circulating neutrophil count of less than 500 µL.^44^ The most common clinical course of agranulocytosis is associated with pharyngotonsillitis, stomatitis and/or pneumonia. The frequency of the disease varies with age, with only 10% of cases being reported in children and young adults, and more than half of the episodes occurring in people over 60 years of age. It is a rare condition and is associated with a fatality rate of 8%–10%. Association rates with drug use vary in the different studies, but in Brazil, for example, it is around 56%.^45^

No randomized clinical trials using dipyrone as a postoperative analgesic in children have reported the occurrence of agranulocytosis to date. Studies show that the incidence of agranulocytosis varies between countries. The LATIN^45^ study was a prospective case-control study carried out in cities in Brazil, Argentina and Mexico. The overall incidence rate was estimated at 0.38 per million inhabitants/year. Methimazole was the only drug significantly associated with agranulocytosis (*p* < 0.001), and there was no significant association with dipyrone. A rare incidence of agranulocytosis associated with drugs has been reported in a retrospective study in the city of São Paulo/Brazil (0.44 to 0.82 cases per million inhabitants/year)^46^ and in the collaborative study of Brazil, Argentina and Mexico mentioned above, similar to the finding in Thailand – 0.8:1 million inhabitants/year.^47^ This contrasts with the higher incidence reported in the United States of America, from 2.4 to 15.4 per million inhabitants/year,^48^ and in European countries, such as that of the collaborative study in Germany, Italy, Spain, Hungary, Bulgaria and Sweden, in addition to Israel, which found 1.1–6.2 cases/million/year and a mortality rate of 0.5 cases/million/year. However, this study noted a great regional variability in the presentation of blood dyscrasias.^49^ The risk was significantly associated with the use of ticlopidine, sulphonamides, non-steroidal anti-inflammatory drugs, calcium dobesilate, antithyroid drugs, spironolactone and dipyrone. There was a subsequent study, in the city of Barcelona, which found an incidence of agranulocytosis associated with medications in the order of 3.46 cases per million inhabitants/year.^20^ In France, the corresponding number was 6 cases per million inhabitants/year. The specific incidence of agranulocytosis associated with dipyrone varied from one for every 1,439 prescriptions in Sweden,^36^ to 0.56 cases per million inhabitants/year in Barcelona,^20^ reaching up to 0.96 cases per million/year in Berlin.^19^ A cohort of hospitalized patients in Bogotá, Colombia, involving 2,743 patients, showed no cases of agranulocytosis. In the LATIN study,^46^ the Odds Ratio (OR) for drug-associated agranulocytosis was 2.4 (95% CI 0.8–6.7). The corresponding figures in Barcelona were 25.8 (95% CI 8.8–79.1).^20^ In a study published in 2020, the OR for agranulocytosis and drug-induced neutropenia was 3.03 (95% CI 2.49–3.69). The risk of developing agranulocytosis and neutropenia after a dipyrone prescription was 1:1,602 (95% CI 1:1,926–1:1,371). There are several possible explanations for the differences found in incidence between the various studies, ranging from the use of different methodologies to the genetic heterogeneity of populations, with probable gene polymorphisms of their own, which have not yet been studied specifically for dipyrone.

Even though the risk of agranulocytosis with dipyrone is undeniable, its real incidence in the population is not known, but it is assumed to be low. For this reason, the German consensus that brought together several representative entities concluded that dipyrone has a positive risk-benefit rate compared to other non-opioid analgesics, recommending its use.

### Other adverse effects (gastrointestinal, cardiovascular and anaphylaxis)

Non-severe conditions include nausea, vomiting, epigastric pain, dry mouth, asthenia, rash, and hypotension without syncope. Anaphylaxis, reactions similar to asthma, hemodynamic collapse, serum sickness, Stevens- Johnson syndrome, vasculitis, alveolitis, pneumonitis, hepatitis, hemolytic-uremic syndrome and agranulocytosis are considered severe. All the data discussed here are valid for short-term use, no longer than two weeks (usually up to 7 days), as there are no studies analyzing the occurrence of adverse effects with the medium and long-term use of dipyrone.

The incidence rate of adverse effects to dipyrone varies between countries. Itching, edema, and rash were reported in one patient each (total 0.3%, 95% CI 0.035–0.56) from a sample of 1177 children under 6 years of age in 6 German pediatric centers which used perioperative dipyrone. There were no adverse effects on heart rate and blood pressure, as well as on breathing, and the study was unable to detect any cases of agranulocytosis.^22^ In the Colombian cohort of hospitalized patients,^50^ a global incidence of adverse effects was found in the order of 0.3% (7/2743), which can translate into 0.5/1000 person-days or 0.14 cases/1000 applied doses. In other words, incidence of 1 case every 1979 person-days or 6928 person-doses. In 100% of the cases, the adverse reactions reported were skin reactions, which cleared with the discontinuation of the drug and with specific therapy. There was neither admission to the ICU nor mortality associated with dipyrone in this study.^50^ In all clinical trials of dipyrone use in the postoperative period of children, the incidence of nausea and vomiting was the same between the active group and the placebo.

There are several case reports describing anaphylaxis after administration of the drug.^51^ For most non-opioid analgesics, the incidence of anaphylaxis is in the range of 5–15 cases/100,000 exposed patients.^52^ Estimates range from 2.1/100,000 for oral aspirin to 16/100,000 for diclofenac suppository, with oral or parenteral dipyrone having an intermediate incidence between 7 and 8 episodes/100,000 patients.^52^ It is a lower incidence than with parenteral penicillin (32/100,000) or with radiological contrast media (71/100,000), for example.^52^ It should be noted that there is anaphylaxis described with the use of other common painkillers as well, such as aspirin and ibuprofen.^53^ Meta- analysis that compared the outcome of adverse effects between short-term use of dipyrone and other analgesics, involving studies with almost 4000 participants combined, showed no significant difference between them (relative risk 0.91, 95% CI 0.79–1.05).^54^ Another study using the World Health Organization's pharmacovigilance database (VigiBase – https://tools.Who-umc.org/webroot/ [access restricted]) that includes data from 110 countries and more than 10 million reports of individual cases, demonstrated that dipyrone was even safer than other non-steroidal analgesics for the gastrointestinal tract and kidneys. The risk for developing duodenal or gastric ulcers was 0.9 (95% CI 0.7–1.2) for dipyrone and 14.3 (95% CI 13.8–14.9) for diclofenac, for example. The corresponding numbers for upper digestive bleeding were 1.5 (1.3–1.7) and 9.1 (8.8–9.3), respectively. As for the decline in renal function, the difference in favor of dipyrone was smaller, but still significant, with an estimated relative risk for dipyrone was 1.3 (1.0–1.4) and 2.3 (2.2–2.4) for diclofenac.^33^ It has been shown that the lower incidence of digestive bleeding with dipyrone is the main contributing factor to the lower risk of fatal adverse effects with dipyrone compared to diclofenac (25 vs. 592 per 100 million users).^55^

Although mildly symptomatic hypotension may occur in a few patients, without hemodynamic repercussions, it is possible that severe hypotension may also occur, even without an allergic reaction. When intravenous dipyrone was administered, in 7 (0.34%) of 2.053 patients there was a decrease in systolic pressure of at least 20 mmHg, occurring within minutes up to 6 h.^56^

## Conclusion

Although it appears that dipyrone exhibits a profile suitable for use in the postoperative period following tonsillectomy in children, as demonstrated in clinical trials dealing with abdominal and other surgeries in these age group, more well-designed studies are needed to establish its role in the postoperative period after tonsillectomy due to the scarcity of randomized clinical trials evaluating its postoperative analgesic effect. The argument for the occurrence of agranulocytosis does not seem strong enough to justify the abandonment of these studies, because its incidence is very low, mainly in children, at least in Latin America. Specifically in low-income countries, its use is attractive because it has a low cost and can be used intravenously, an advantageous feature in the postoperative period of tonsillectomy where odynophagia tends to be severe and universal.

## Conflicts of interest

The authors declare no conflicts of interest.

## References

[1] Apfelbaum J.L., Ashburn M.A., Connis R.T., Gan T.J., Nickinovich D.G., Caplan R.A. (2012). Practice guidelines for acute pain management in the perioperative setting: an updated report by the American Society of Anesthesiologists Task Force on Acute Pain Management. Anesthesiology.

[2] Kissin I. (2000). Preemptive analgesia. Anesthesiology.

[3] Boric K., Dosenovic S., Kadic A.J., Batinic M., Cavar M., Urlic M. (2017). Interventions for postoperative pain in children: an overview of systematic reviews. Paediatr Anaesth.

[4] Parrilla E.M.C., Camarasa B.G., Dueñas C.G., Liceras E.L., Sattuf K.M., Peinado G.C. (2018). Comparative study of the effect of two analgesic techniques in postoperative pain control in pediatric surgery. Cir Pediatr.

[5] Caliskan E., Sener M., Kocum A., Ozyilkan N.B., Ezer S.S., Aribogan A. (2013). The efficacy of intravenous paracetamol versus dipyrone for postoperative analgesia after daycase lower abdominal surgery in children with spinal anesthesia: a prospective randomized double-blind placebo-controlled study. BMC Anesthesiol.

[6] Kocum A.I., Sener M., Caliskan E., Bozdogan N., Micozkadioglu D., Yilmaz I. (2013). Intravenous paracetamol and dipyrone for postoperative analgesia after day-case tonsillectomy in children: a prospective, randomized, double blind, placebo controlled study. Braz J Otorhinolaryngol.

[7] Sener M., Kocum A., Caliskan E., Yilmaz I., Caylakli F., Aribogan A. (2015). Administration of paracetamol versus dipyrone by intravenous patient-controlled analgesia for postoperative pain relief in children after tonsillectomy. Rev Bras Anestesiol.

[8] Schärli A.F., Brulhart K., Monti T. (1990). Pharmacokinetics and therapeutic study with nimesulide suppositories in children with post-operative pain and inflammation. J Int Med Res.

[9] Cuevas H.M., Fernandez M.C.L., Segura R.T.M., de la Rosa J.E.Z., Espino E.Z. (1997). Control del dolor con Ketorolac em niños postoperados de cirugía oftalmológica. Rev Mex Anest.

[10] Penuelas-Acuña J., Oriol-Lopez S.A., Hernandez-Bernal C.E., Castelazo A. (2003). Ketorolac vs. metamizol pre-emptive analgesia in children. Cir Cir.

[11] Lauretti G.R., Dos Reis M.P., De Mattos A.L., Cardia M.C. (1999). Multimodal preventive analgesia in children undergoing inguinal herniorrhaphy under caudal blockage with bupivacaine. Effectiveness of the use of ketamine and dipyrone. Rev Soc Esp Dolor.

[12] Witschi L., Reist L., Stammschulte T., Erlenwein J., Becke K., Stamer U. (2019). Perioperative use of metamizole and other nonopioid analgesics in children: results of a survey. Anaesthesist.

[13] Reist L., Erlenwein J., Meissner W., Stammschulte T., Stüber F., Stamer U.M. (2018). Dipyrone is the preferred nonopioid analgesic for the treatment of acute and chronic pain. A survey of clinical practice in German-speaking countries. Eur J Pain.

[14] Faponle P.A.F., Soyannwo A.O., Ajavi I.O. (2001). Postoperative pain therapy: a survey of prescribing patterns and adequacy of analgesia in Ibadan, Nigeria. Cent Afr J Med.

[15] Matus C.A.B., Zúniga R.M.F. (2011). Errors in managing postsurgical pediatric pain in Mexico. J Pain Palliat Care Pharmacother.

[16] Curtis B.R. (2014). Drug-induced immuneneutropenia/agranulocytosis. Immunohematology.

[17] Fieler M., Eich C., Becke K., Badelt G., Leimkühler K., Messroghli L. (2015). Metamizole for postoperative pain therapy in 1177 children: a prospective, multicentre, observational, postauthorisation safety study. Eur J Anaesthesiol.

[18] International Agranulocytosis and Aplastic Anemia Study Risks of agranulocytosis and aplastic anemia (1986). A first report of their relation to drug use with special reference to analgesics. JAMA.

[19] Huber M., Andersohn F., Sarganas G., Bronder E., Klimpel A., Thomae M. (2015). Metamizole-induced agranulocytosis revisited: results from the prospective Berlin Case-Control Surveillance Study. Eur J Clin Pharmacol.

[20] Ibanez L., Vidal X., Ballarin E., Laporte J.R. (2005). Agranulocytosis associated with dipyrone (metamizol). Eur J Clin Pharmacol.

[21] Rollason V., Desmeules J.A. (2015). Use of metamizole in children and the risk of agranulocytosis Is the benefit worth the risk?. Eur J Anaesthesiol.

[22] Souki M.A. (2016). Metamizole for postoperative pain therapy. Eur J Anaesthesiol.

[23] Kraemer W.F. (2010). Treatment of acute pediatric pain. Semin Pediatr Neurol.

[24] Arrais O.S., Fernandes M.E.P., Dal Pizzol T.S., Ramos L.R., Mengue S.S., Luiza V.L. (2016). Prevalence of self-medication in Brazil and associated factors. Rev Saude Publica.

[25] Cullen K.A., Hall M.J., Golosinskiy A. (2009). Ambulatory surgery in the United States, 2006. Division of health care statistics. Natl Health Stat Report.

[26] Hall M.J., Schwartzman A., Zhang J., Liu X. (2017). Ambulatory surgery data from hospitals and ambulatory surgery centers: United States, 2010. Natl Health Stat Report.

[27] Mitchell R.B., Archer S.M., Ishman S.L., Rosenfeld R.M., Coles S., Finestone S.A. (2019). Practice guideline: tonsillectomy in children (update). Otolaryngol Head Neck Surg.

[28] U.S Food and Drug Administration (2013). FDA drug safety communication: safety review update of codeine use in children; new Boxed Warning and Contraindication on use after tonsillectomy and/or adenoidectomy. Issued. https://www.fda.gov/downloads/Drugs/DrugSafety/UCM339116.pdf.

[29] Santos C.M.D.C., Pimenta C.A.D.M., Nobre M.R.C. (2007). A estratégia PICO para a construção da pergunta de pesquisa e busca de evidências. Rev Latino-Am Enfermagem.

[30] Azevedo C.B., Carenzi L.C., Queiroz D.L.C., Anselmo-Lima W.T., Valera F.C.P., Tamashiro E. (2014). Clinical utility of PPPM and FPS-R to quantify post-tonsillectomy pain in children. Int J Pediatr Otorhinolaryngol.

[31] Stern J.A.C., Savovic J., Page M.J., Elbers R.G., Blencowe N.S., Boutron I. (2019). RoB 2: a revised tool for assessing risk of bias in randomized trials. BMJ.

[32] Shamseer L., Moher D., Clarke M., Ghersi D., Liberati A., Petticrew M. (2015). The PRISMA-P Group. Preferred Reporting Items for Systematic Review and Meta-Analysis Protocols (PRISMA-P) 2015: elaboration and explanation. BMJ.

[33] Konijnenbelt-Peters J., Van der Heijden C., Ekhart C., Bos J., Bruhn J., Kramers C. (2017). Metamizole (Dipyrone) as an alternative agent in postoperative analgesia in patients with contraindications for nonsteroidal anti-inflammatory drugs. Pain Pract.

[34] de Leeuw T.G., Dirckx M., Gonzalez Candel A., Scoones G.P., Huygen F.J.P.M., de Wildt S.N. (2017). The use of dipyrone (metamizol) as an analgesic in children: what is the evidence? A review. Paediatr Anaesth.

[35] Hamunen K., Kontinen V. (2005). Systematic review on analgesics given for pain following tonsillectomy in children. Pain.

[36] Cho H.K., Kim K.W., Jeong Y.M., Lee H.S., Hwang S.H. (2014). Efficacy of ketamine in improving pain after tonsillectomy in children: meta-analysis. PLoS One.

[37] Tong Y., Ding X.-B., Wang X., Ren H., Chen Z.X., Li Q. (2014). Ketamine peritonsillar infiltration during tonsillectomy in pediatric patients: an updated meta-analysis. Int J Pediatr Otorhinolaryngol.

[38] Afman C.E., Welge J.A., Steward D.L. (2006). Steroids for post-tonsillectomy pain reduction: meta-analysis of randomised controlled trials. Otolaryngol Head Neck Surg.

[39] Steward D.L., Grisel J., Meinzen-Derr J. (2011). Steroids for improving recovery following tonsillectomy in children. Cochrane Database Syst Rev.

[40] Sun J., Wu X., Meng Y., Jin L. (2010). Bupivacaine versus normal saline for relief of post- adenotonsillectomy pain in children: a meta-analysis. Int J Pediatr Otorhinolaryngol.

[41] He X.-Y., Cao J.-P., Shi X.-Y., Zhang H. (2013). Dexmedetomidine versus morphine or fentanyl in the management of children after tonsillectomy and adenoidectomy: a meta-analysis of randomised controlled trials. Ann Otol Rhinol Laryngol.

[42] Hobson A., Wiffen P.J., Conlon J.A. (2015). As required versus fixed schedule analgesic administration for postoperative pain in children. Cochrane Database Syst Rev.

[43] Fedorowicz Z., van Zuuren E.J., Nasser M., Carter B., Al Langawi J.H. (2013). Oral rinses, mouthwashes and sprays for improving recovery following tonsillectomy. Cochrane Database Syst Rev.

[44] Andrès E., Maloisel F. (2008). Idiosyncratic drug-induced agranulocytosis or acute neutropenia. Curr Opin Hematol.

[45] Hamerschlak N., Maluf E., Cavalcanti A.B., Junior A.A., Eluf-Neto J., Falcão R.P. (2008). Incidence and risk factors for agranulocytosis in Latin American countries – the Latin Study, a multicenter study. Eur J Clin Pharmacol.

[46] Hamerschlak N., Montezuma M.P., Bacal N., Szterling L.N., Rosenfeld L.G., Guerra C.C. (1993). Retrospective prevalence and incidence of drug-induced agranulocytosis in the city of São Paulo-Brasil. Rev Paul Med.

[47] Shapiro S., Issaragrisil S., Kaufman D.W., Anderson T., Chansung K., Thamprasit T. (1999). Agranulocytosis in Bangkok, Thailand: a predominantly drug-induced disease with an unusually low incidence. Aplastic Anemia Study Group. Am J Trop Med Hyg.

[48] Strom B.L., Carson J.L., Schinnar R., Snyder E.S., Shaw M. (1992). Descriptive epidemiology of agranulocytosis. Arch Intern Med.

[49] (1986). Risks of agranulocytosis and aplastic anemia. A first report of their relation to drug use with special reference to analgesics. The International Agranulocytosis and Aplastic Anemia Study. JAMA.

[50] González-Cárdenas V.H., Laverde-Gaonad L.A., Cabarique-Serrano S.H., Cháves- Rojasa N., Reina-Sierra J.Á, Infante J.S. (2018). Analysis of the incidence of adverse events related to the administration of dipyrone. Rev Colomb Anestesiol.

[51] Stamer U.M., Soehlea M., Park T.W., Fischer M., Stuber F. (2007). Anaphylactic reaction after intravenous dipyrone. Acute Pain.

[52] Kaufman D.W., Kelly J.P. (2003). The International Collaborative Study of severe anaphylaxis risk of anaphylaxis in a hospital population in relation to the use of various drugs: an international study. Pharmacoepidemiol Drug Saf.

[53] Bellegrandi S., Rosso R., Mattiacci G., Zaffiro A., Di Sora F., Menzella F. (1999). Combined immediate-and delayed-type hypersensitivity to metamizole. Allergy.

[54] Kötter T., Da Costa B.R., Fässler M., Blozik E., Linde K., Jöni P. (2015). Metamizole-associated adverse events: a systematic review and meta-analysis. PLoS One.

[55] Andrade S.E., Martinez C., Walker A.M. (1998). Comparative safety evaluation of non- narcotic analgesics. J Clin Epidemiol.

[56] Zoppi M., Hoihn’e R., Keller M.F., Streit F., Hess T. (1983). Blutdruckabfall unter Dipyrone. Schweiz Med Wochenschr.

